# Preoperative symptoms of depression, anxiety, and cognitive impairment in glioma patients: A cerebral perfusion CT study

**DOI:** 10.1002/brb3.3020

**Published:** 2023-05-01

**Authors:** Ke Wang, Wanrui Fu, Shenjie Li, Lizhen Chen, Yajie Gan, Wei Xiang, Ligang Chen, Jie Zhou

**Affiliations:** ^1^ Key Laboratory of Advanced Technologies of Materials, Ministry of Education, School of Materials Science and Engineering Southwest Jiaotong University Chengdu China; ^2^ Department of Neurosurgery The Affiliated Hospital of Southwest Medical University Luzhou China; ^3^ Huadong Hospital Fudan University Shanghai China; ^4^ Neurosurgery Clinical Medical Research Center of Sichuan Province Luzhou China; ^5^ Neurological Diseases and Brain Function Laboratory Luzhou China; ^6^ Department of Clinical Laboratory Chengdu Women's and Children's Central Hospital Chengdu China

**Keywords:** anxiety, cognitive impairment, depression, glioma, imaging

## Abstract

**Purpose:**

Glioma patients have varying degrees of psychiatric symptoms, which severely affect the quality of life of patients and their families. The present study investigated the correlation between preoperative psychiatric symptoms and local cerebral perfusion parameters of in glioma patients.

**Patients and methods:**

The depression, anxiety, and cognitive impairment (CI) scores of 39 patients were assessed separately, and all of the patients underwent a preoperative perfusion computed tomography scan.

**Results:**

This study found that: (1) The incidence of preoperative symptoms of depression, anxiety, and CI was 46.15%, 48.72%, and 25.64%, respectively. (2) Cerebral blood volume (CBV) (lesion‐sided [LS] occipital lobe white matter [WM] and parietal lobe WM and normal‐sided temporal lobe WM), permeability surface (PS) (LS temporal lobe gray matter [GM] and parietal lobe WM) in the depression group were significantly decreased (*p* < .05). (3) CBV (LS occipital lobe WM), cerebral blood flow (LS parietal lobe GM, centrum ovale and frontal lobe WM and normal‐sided frontal lobe WM, temporal lobe WM and parietal lobe WM), and mean transition time (MTT) (normal‐sided frontal lobe WM and temporal lobe WM) in the anxiety group were significantly increased (*p* < .05). (4) CBV (LS temporal lobe GM), MTT (LS anterior limb of internal capsule), and PS (LS thalamus) in the CI group were significantly increased (*p* < .05).

**Conclusion:**

This study showed that glioma patients had different levels of psychological distress in glioma patients before surgery, which may be related to the changes in brain perfusion caused by the tumor.

## Graphical abstract

Patients with glioma were often accompanied by complications such as depression, anxiety, cognitive impairment (CI) and personality changes, which had a serious impact on the quality of life of patients and their families. This study was the first to analysis the perfusion parameters and pre‐operative symptoms of depression, anxiety and CI in patients with glioma. The findings of our study were: 1) patients with glioma could have pre‐opertive symptoms of depression, anxiety and CI; 2) preoperative symptoms of depression, anxiety and CI in patients with glioma could be closely with changes of cerebral perfusion, either on the lesion side or on the non‐lesion side. 3) the presence of tumor could affect cerebral blood perfusion, which could be one of the pathophysiological factors of preoperative psychiatric symptoms in patients with glioma.

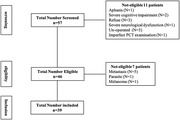

**Abbreviations**
BBB: blood–brain barrier
CBF: cerebral blood flow
CBV: cerebral blood volume
CN: cognitive normality
CI: cognitive impairment
CT: computed tomography
GM: gray matter
KPS: Karnofsky Performance Status
NLS: non‐lesion side
LS: lesion side
MRI: magnetic resonance imaging
MTT: mean transition time
PCT: perfusion computed tomography
PS: permeability surface
ROIs: regions of interest
TTP: time to peak
WM: white matter

## 1 INTRODUCTION

Clinicians often focus on the symptoms of intracranial hypertension and neurological function, such as headache, epilepsy, and hemiparesis in glioma patients, but the psychological symptoms, such as depression, anxiety, and cognitive impairment (CI), were easily ignored (Aldape et al., 2019; Lapointe et al., 2018; Rooney et al., 2014). Previous studies have shown that glioma patients suffer from varying degrees of depression (15%–50%), anxiety (30%–63%), and CI (29%–90%), which can seriously affect the patients’ quality of life and survival time (D'Angelo et al., 2008; Rooney et al., 2014; van Loon et al., 2015; Zwinkels et al. 2016). In addition, the onset of psychiatric symptoms may be the initial trigger for clinical presentation in several cases of the glioblastoma multiforme (Leo et al., 2020). Previous studies have suggested that symptom manifestation is likely to be influenced by tumor location and size, tumor‐induced increases in intracranial pressure, functional disturbances in transmission and connectivity of neural pathways caused by gray and/or white matter (GM/WM) compression, and redistribution of cerebral blood flow (CBF) caused by vascular compression (Silvani et al., 2011). However, the pathophysiological mechanism remains unclear.

Studies have shown that abnormalities in brain structure and function caused by changes in CBF perfusion may be a major cause of psychiatric symptoms. In the study of primary depression, Taylor et al. (2013) proposed the “vascular depression hypothesis,” which links underlying vascular risk factors to adverse effects on brain function that influence the development of depression. Meanwhile, some studies have found reduced perfusion in different brain regions in patients with mild CI or Alzheimer's disease (Binnewijzend et al., 2013; Dai et al., 2009; Lou et al., 2016). In Alzheimer's disease and Parkinson's disease, some studies have shown that positron emission tomography‐computed tomography (CT) can detect brain metabolism decline and correlate the severity of psychiatric symptoms with metabolic changes (Carey et al., 2021; Henderson et al., 2020; Wolinsky et al., 2018). The changes in cerebral perfusion caused by various factors appear to play an important role in the development of psychiatric symptoms. Meanwhile, tumor formation and growth are accompanied by abnormal biological metabolism and angiogenesis, leading to obvious changes in blood flow in tumors, peritumoral brain regions, and even non‐lesional lateral brain regions (Boele et al., 2015; Fan et al., 2019; Mugge et al., 2020). Whether abnormalities in CBF play an important role in glioma‐associated psychological distress is a matter for further study.

In the present study, we investigated tumor perfusion and its relationship with perfusion in the normal brain regions using perfusion computed tomography (PCT). Second, the relationship between perfusion parameters of brain regions and psychiatric symptoms was investigated.

## 2 MATERIALS AND METHODS

### 2.1 Subjects

**FIGURE 1:**
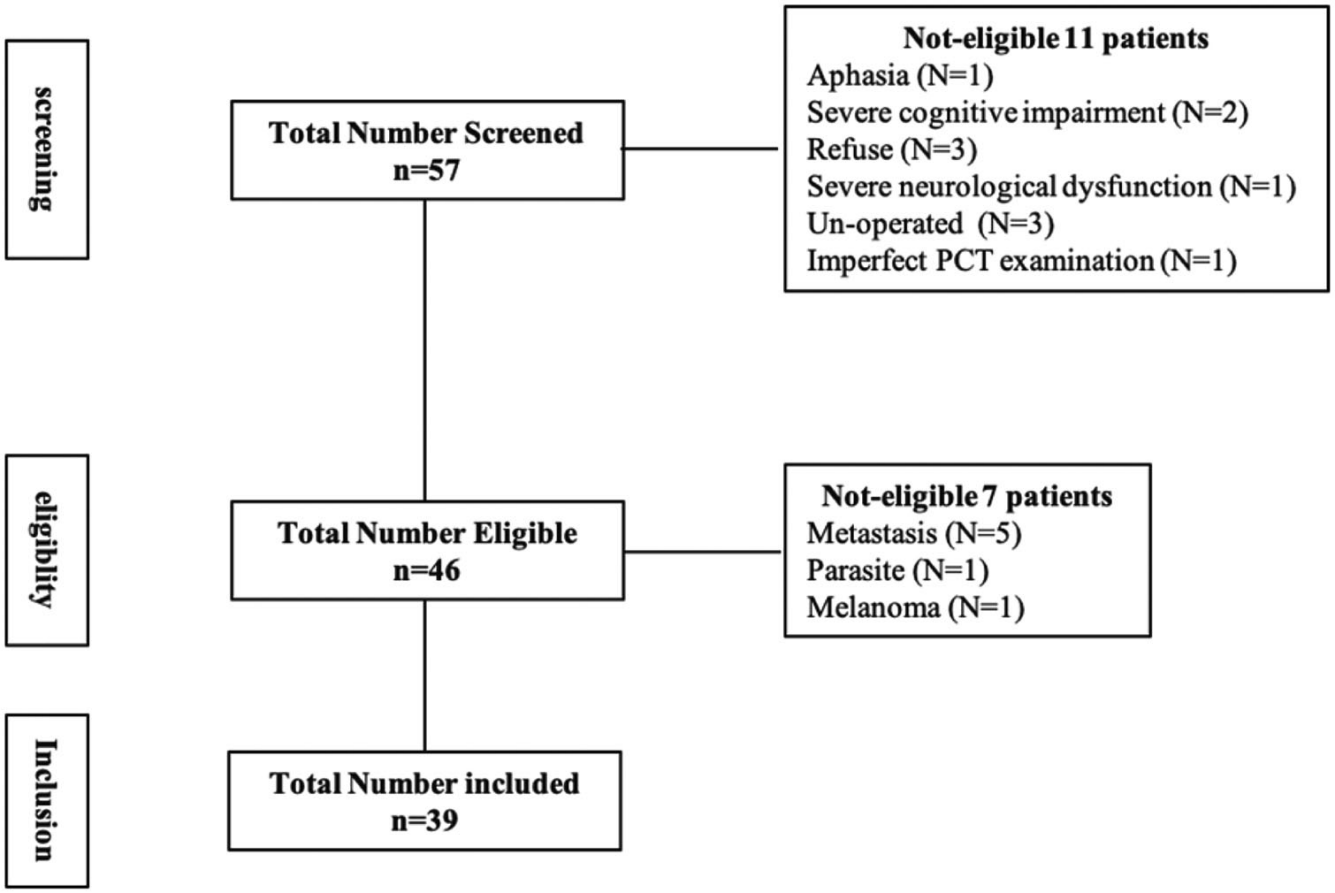
Flowchart of the study selection process.

We reviewed the records of 57 consecutive patients who underwent preoperative PCT for newly diagnosed glioma at the Department of Neurosurgery, the Affiliated Hospital of Southwest Medical University from January 2018 to November 2018. The inclusion criteria were as follows: (1) had complete clinical data, including preoperative PCT and psychiatric assessment; (2) pathologically confirmed glioma; (3) education beyond primary school; (4) patients who volunteered for research and the psychiatric assessment were eligible; (5) right‐handedness. Exclusion criteria were as follows: (1) patients with recurrent glioma; (2) previous brain biopsy or surgery; (3) unable to participate in the psychiatric assessment; (4) had a medical history or family history of psychological distress. Finally, 39 cases were included (Figure 1), including 19 males and 20 females, the age range from 15 to 83‐year old, mean (49.08 ± 14.74) years. The postoperative pathological diagnosis was glioma, including WHO I grade 3 cases, WHO II grade 12 cases, WHO III grade 10 cases, and WHO IV grade 14 cases (Table 1).

**TABLE 1:** Baseline characteristics of the glioma patients

Characteristics	No. (39)	Proportion (%)
Ages (years), (mean ± SD)	49.08 ± 14.74	
Gender (male/female)	19/20	48.7/51.3
Tumor grade		
WHO I	3	7.7
WHO II	12	30.8
WHO III	10	25.6
WHO IV	14	35.9
Location		
Frontal lobe	15	38.5
Temporal lobe	7	17.9
Frontotemporal lobe (including insula)	4	10.3
Parietal lobe	3	7.7
Occipital lobe	4	10.3
Thalamus and basic ganglion	2	5.1
Cerebellum	4	10.3
Side		
Right	16	41.0
Left	19	48.7
Bilateral	4	10.3
Education		
Primary school	22	56.4
Junior middle school	8	20.5
High school	5	12.8
University and above	4	10.3
Marriage		
Married	36	92.3
Unmarried	3	7.7
KPS score		
≤80	13	33.3
>80	26	66.7

Abbreviation: KPS, Karnofsky Performance Status.

### 2.2 PCT protocol

The method of the PCT protocol was based on our previous study (Wang et al., 2021). First, all patients underwent a non‐contrast‐enhanced CT scan on the Philips Brilliance 256‐slice spiral CT scanner after the patients had undergone an iodine anaphylaxis test and the result was negative. Second, the contrast agent (iobitridol, 350 mgI/mL) was administered rapidly (6 mL/s) via an elbow intravenous bolus injection at the elbow using an automatic injector (2 mL/kg). Third, normal saline (30 mL) was injected at the same rate. After a delay of 5 s, scanning was performed with the following parameters: 80 kV, 100 mA s, 0.4 s/cycle, 4.1 s interval, 13 cycles in total, 5 mm slice thickness, 512 × 512 matrix, 54.4 s contrast agent tracking time, and 12.8 cm coverage. Finally, the reconstructed dynamic images were transferred to the workstation for processing in the Philips Extended Brilliance Workstation using CT brain perfusion software.

### 2.3 Selection of regions of interest (ROIs)

The regions of interest (ROIs) included the GM and WM of the bilateral frontal, parietal, occipital, and temporal lobes, as well as the bilateral internal capsule, thalamus, hippocampus, centrum semiovale, and the parenchyma of tumor. The ROIs of the bilateral frontal lobe, thalamus, temporal lobe, occipital lobe, bilateral internal capsule were selected at the level of the basal ganglia. The ROIs of the bilateral hippocampus were selected at the maximal level of the hippocampal axis. The ROIs of the bilateral centrum semiovale were selected at the first level upward from the top of the lateral ventricle. The ROIs of the bilateral parietal lobes were selected at the second level from the centrum semiovale upward. The glioma ROIs were selected at multidimensional parenchymal areas of the tumors, and the final perfusion parameters of the tumors were averaged (Figure S1). The ROIs were divided into lesion side (LS) and non‐lesion side (NLS). For bilateral and subtentorial tumors, the dominant hemisphere side was the LS and the contralateral side was the NLS. We did not measure the affected ROIs that were closed by the tumor or edema, or where the anatomical structures were difficult to distinguish.

### 2.4 PCT data processing and analysis

Two experienced radiologists, blinded to the results of the psychiatric assessment, were responsible for measuring perfusion parameters. A third radiologist participated in the evaluation if two radiologists were in disagreement. For supratentorial tumors, the afferent artery was the ascending petrous segment of the internal carotid artery. For subtentorial tumors, the afferent artery was the basilar artery. The efferent vein was the superior sagittal sinus. Combined with the preoperative magnetic resonance imaging (MRI) of the patients, the radiologist and neurosurgeon manually drew ROIs, avoiding the necrotic or cystic parts of the tumor and cortical vessels, to generate the time density curve, the false‐color images and perfusion parameters of ROIs, including CBF, cerebral blood volume (CBV), mean transition time (MTT), time to peak (TTP), and permeability surface (PS). The parameter values of the ROIs were corrected by the value of hematocrit.

### 2.5 Neuropsychological assessment

Depression was assessed using the 17‐item Hamilton Depression Rating Scale, with scores ≥7 indicating symptoms of depression (Hamilton, 1960). Anxiety was assessed using the Hamilton Anxiety Scale, with scores >7 indicating symptoms of anxiety (Hamilton, 1959). CI was assessed using the Mini Mental State Examination, with the symptoms of CI were defined as ≤24 points for junior high school education and above, ≤20 points for primary school education, and ≤17 points for illiteracy, respectively (Folstein et al., 1975). All interviews were scored by a clinically experienced psychiatrist.

### 2.6 Statistical analyses

SPSS 22.0 statistical software was used for statistical analysis. The number of count data cases (percentage) was expressed, and the measurement data were expressed as mean ± standard deviation (*x* ± *s*). Measurement data were analyzed by independent samples *t*‐test, and count data were analyzed by chi‐squared test or rank‐sum test. Pearson's correlation analysis was used for correlation analysis. *p* ≤ .05 was considered statistically significant.

## 3 RESULTS

### 3.1 Clinical characteristics

**FIGURE 2:**
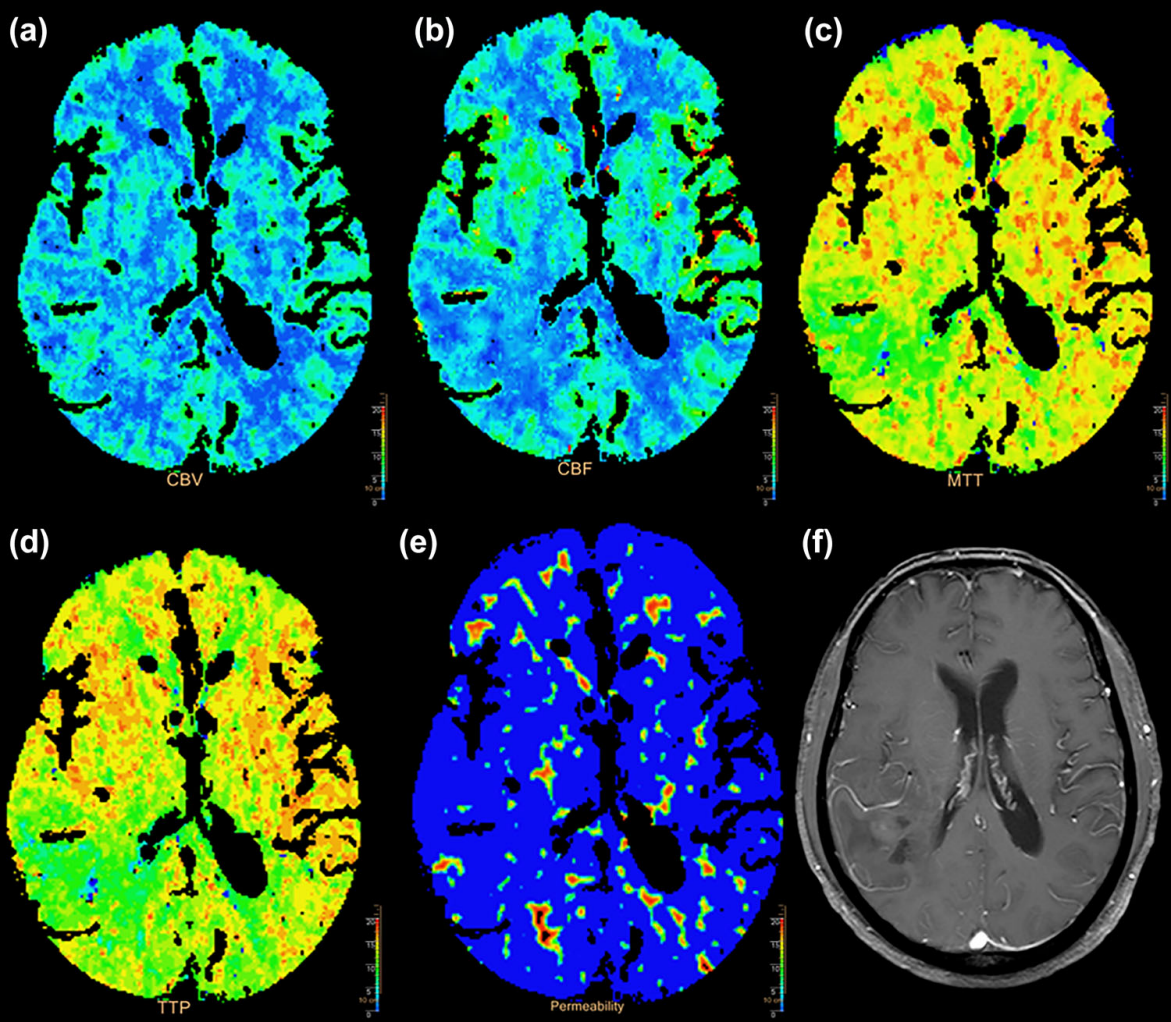
60‐year‐old man with an anaplastic astrocytoma of the left frontoparietal lobe, WHO III, normal group: (A) cerebral blood volume (CBV) map; (B) cerebral blood flow (CBF) map; (C) mean transition time (MTT) map; (D) time to peak (TTP) map; (E) permeability surface (PS) map; (F) contrast‐enhanced T1‐weighted magnetic resonance imaging (MRI).

In 39 glioma patients, the numbers of preoperative non‐depression, preoperative mild depression, preoperative moderate depression, and preoperative severe depression were 21 (53.85%), 12 (30.77%), 4 (10.26%), and 2 (5.12%), respectively. Meanwhile, 20 (51.28%) cases had no anxiety before surgery, 13 (33.34%) cases had anxiety before surgery, and 6 (15.38%) cases definitely have anxiety before surgery. Furthermore, the numbers of preoperative cognitive normality (CN) and preoperative CI were 29 (74.36%) and 10 (25.64%), respectively. There were 9 (23.1%) patients in whom all the neuropsychological evaluations were normal (Figure 2). There were no significant differences in terms of age, sex, education level, marital status, operative Karnofsky Performance Status, tumor grade, location, and side in the clinical characteristics between depression and non‐depression groups (Table S1), anxiety and non‐anxiety groups (Table S2), and CI and CN groups (Table S3).

### 3.2 Comparisons of perfusion parameters between depression group and non‐depression group

**FIGURE 3:**
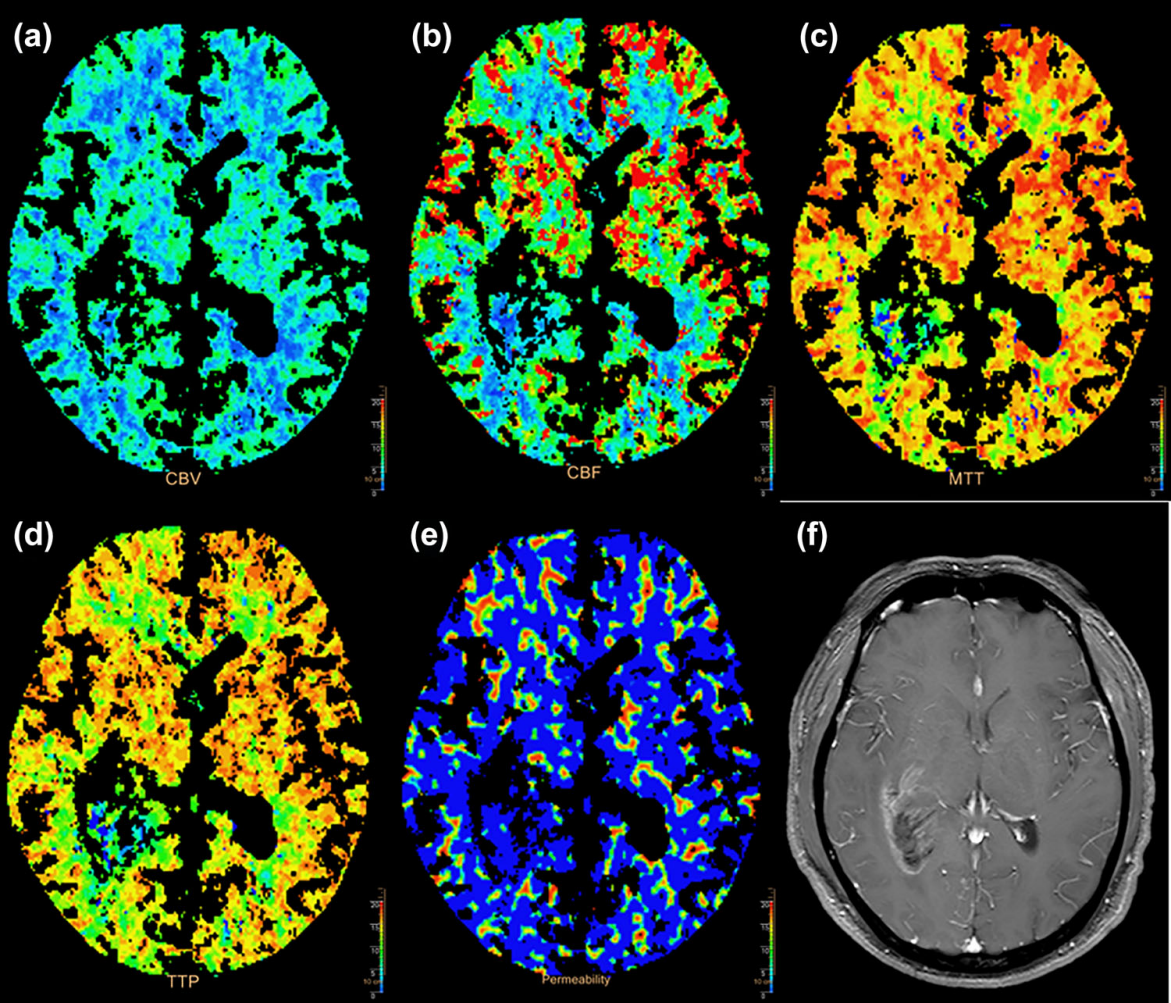
30‐year‐old man with a glioblastoma of the right temporal lobe, WHO IV, depression group: (A) cerebral blood volume (CBV) map; (B) cerebral blood flow (CBF) map; (C) mean transition time (MTT) map; (D) time to peak (TTP) map; (E) permeability surface (PS) map; (F) contrast‐enhanced T1‐weighted magnetic resonance imaging (MRI).

CBV (occipital lobe WM and parietal lobe WM of LS and temporal lobe WM of NLS), PS (temporal lobe GM and parietal lobe WM of LS) in depression group were statistically different compared with that of non‐depression group (Table 2, Figure 3).

**TABLE 2:** The difference in perfusion parameters between the depression group and the non‐depression group

ROIs	Parameter	Non‐depression (*n* = 21)	Depression (*n* = 18)	*t*	*p*
LS temporal GM	PS	14.52 ± 6.01	19.27 ± 5.15	−2.402	.022^*^
LS occipital WM	CBV	7.93 ± 2.04	6.11 ± 2.45	2.282	.030^*^
LS parietal WM	CBV	2.68 ± 0.97	2.09 ± 0.68	2.066	.046^*^
	PS	15.37 ± 3.88	11.72 ± 6.09	2.084	.048^*^
NLS temporal WM	CBV	2.56 ± 0.74	1.96 ± 0.88	2.272	.029^*^

Abbreviations: CBV, cerebral blood volume; GM, gray matter; LS, lesion side; NLS, non‐lesion side; PS, permeability surface; ROIs, regions of interest; WM, white matter.

^*^ *p* < .05.

### 3.3 Comparisons of perfusion parameters between anxiety group and non‐anxiety group

**FIGURE 4:**
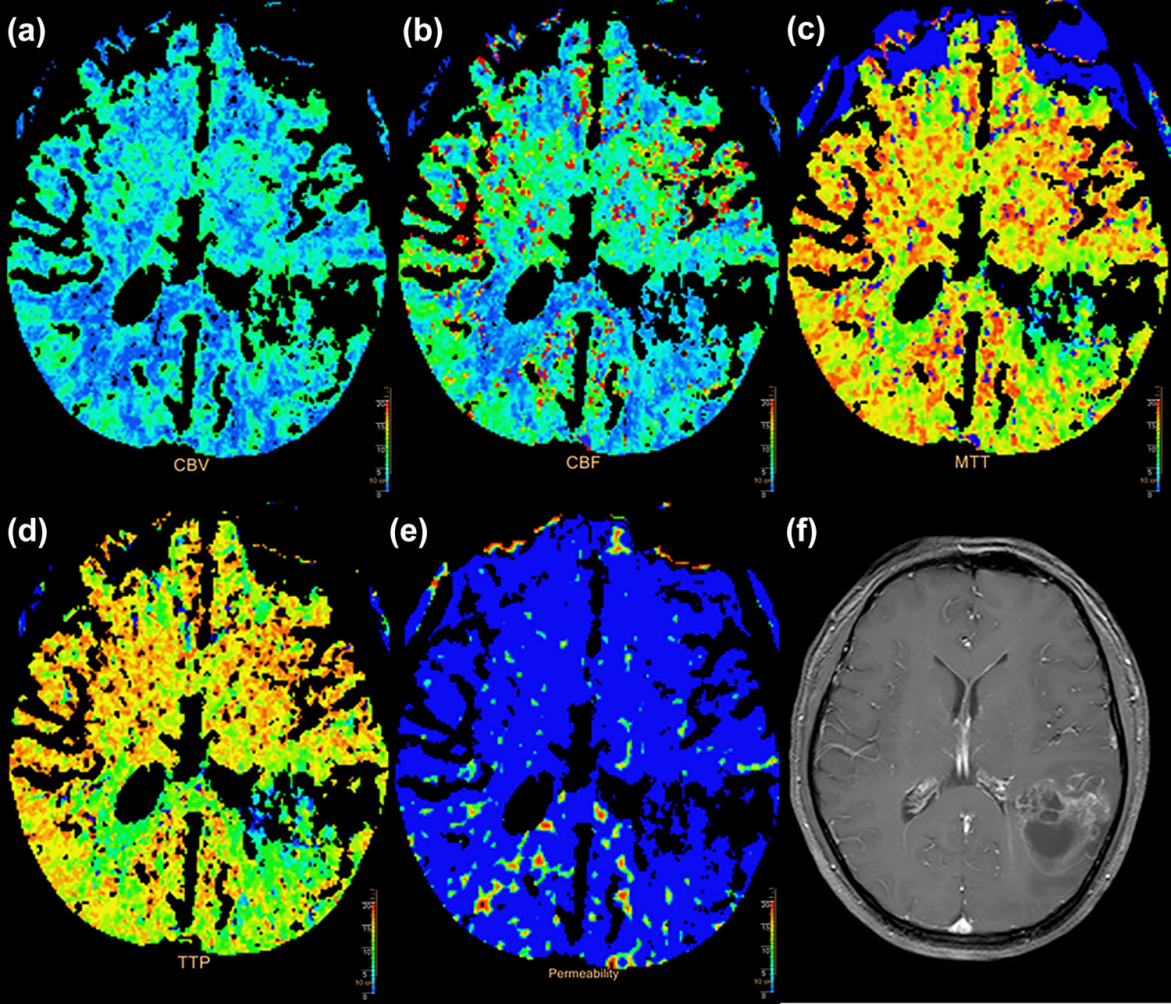
46‐year‐old man with a glioblastoma of the occipital‐temporal lobe, WHO IV, anxiety group: (A) cerebral blood volume (CBV) map; (B) cerebral blood flow (CBF) map; (C) mean transition time (MTT) map; (D) time to peak (TTP) map; (E) permeability surface (PS) map; (F) contrast‐enhanced T1‐weighted magnetic resonance imaging (MRI).

CBV (occipital lobe WM of LS), CBF (parietal lobe GM, centrum ovale and frontal lobe WM of LS and frontal lobe WM, temporal lobe WM and parietal lobe WM of NLS), and MTT (frontal lobe WM and temporal lobe WM of NLS) in anxiety group were statistically different compared with that of non‐anxiety group (Table 3, Figure 4).

**TABLE 3:** The difference in perfusion parameters between the anxiety group and the non‐anxiety group

ROIs	Parameter	Non‐anxiety (*n* = 20)	Anxiety (*n* = 19)	*t*	*p*
LS occipital WM	CBV	2.83 ± 0.73	2.14 ± 0.76	2.610	.014^*^
LS parietal GM	CBF	39.29 ± 23.03	59.22 ± 26.96	−2.395	.022^*^
LS centrum ovale	CBF	19.19 ± 7.17	29.44 ± 18.64	−2.076	.046^*^
LS frontal WM	CBF	21.42 ± 10.15	41.33 ± 33.16	−2.345	.030^*^
NLS frontal WM	CBF	22.02 ± 11.74	38.12 ± 22.30	−2.636	.014^*^
	MTT	7.37 ± 2.23	5.57 ± 2.23	2.318	.027^*^
NLS temporal WM	CBF	20.97 ± 8.77	37.91 ± 18.44	−3.67	.001^*^
	MTT	8.08 ± 4.40	5.75 ± 2.12	2.111	.042^*^
NLS parietal GM	CBF	22.81 ± 9.70	35.29 ± 23.45	−2.134	.043^*^

Abbreviations: CBF, cerebral blood flow; CBV, cerebral blood volume; GM, gray matter; LS, lesion side; MTT, mean transition time; NLS, non‐lesion side; ROIs, regions of interest; WM, white matter.

^*^ *p* < .05.

### 3.4 Comparisons of perfusion parameters between CI group and CN group

**FIGURE 5:**
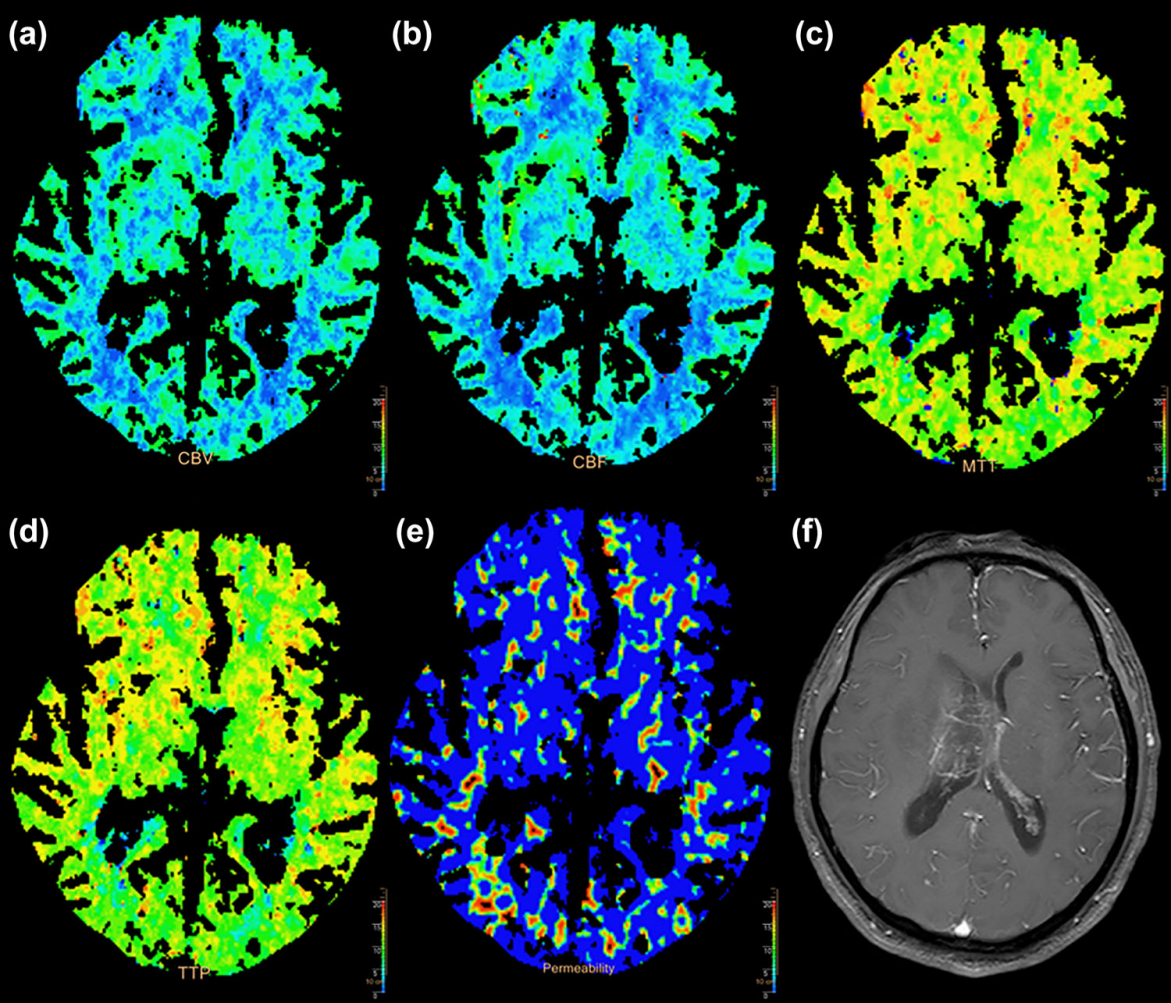
62‐year‐old man with a glioblastoma of the corpus callosum, WHO IV, cognitive impairment (CI) group: (A) cerebral blood volume (CBV) map; (B) cerebral blood flow (CBF) map; (C) mean transition time (MTT) map; (D) time to peak (TTP) map; (E) permeability surface (PS) map; (F) contrast‐enhanced T1‐weighted magnetic resonance imaging (MRI).

CBV (temporal lobe GM of LS), MTT (anterior limb of internal capsule of LS), and PS (thalamus of LS) in CI group were statistically different compared with that of CN group (Table 4, Figure 5).

**TABLE 4:** The difference in perfusion parameters between the cognitive impairment (CI) group and the cognitive normality (CN) group

ROIs	Parameter	CN (*n* = 29)	CI (*n* = 10)	*t*	*p*
LS temporal GM	CBV	4.34 ± 1.38	5.54 ± 1.46	−2.210	.034^*^
LS anterior limb of internal capsule	MTT	4.49 ± 1.51	6.39 ± 2.01	−2.731	.001^*^
LS thalamus	PS	15.78 ± 5.61	20.39 ± 3.93	−2.287	.028^*^

Abbreviations: CBV, cerebral blood volume; GM, gray matter; LS, lesion side; MTT, mean transition time; PS, permeability surface; ROIs, regions of interest.

^*^ *p* < .05

### 3.5 Correlations between tumor parameters and ROIs perfusion parameters of bilateral brain regions

Tumor CBV was positively correlated with LS occipital GM (CBV, MTT, and TTP), LS anterior limb of internal capsule (TTP), LS hippocampus (CBV, MTT, and TTP), LS occipital WM (CBV, TTP), NLS temporal GM (CBV), NLS anterior limb of internal capsule (CBV, MTT, and TTP), NLS posterior limb of internal capsule (CBV, MTT, and TTP), NLS hippocampus (CBV, MTT), NLS parietal GM (TTP), NLS occipital WM (MTT, TTP), and NLS parietal WM (MTT) (Table S4).

Tumor CBF was negatively correlated with LS temporal GM (MMT), LS anterior limb of internal capsule (MTT), LS occipital WM (CBV), and NLS parietal WM (MTT). Furthermore, tumor CBF was positively correlated with LS parietal GM (CBF) (Table S5).

## 4 DISCUSSION

Patients with glioma are often accompanied by complications, such as depression, anxiety, CI, and personality changes, which can seriously impact on the quality of life of patients and their families (Hibar et al., 2018; Shi et al., 2018). At present, there are few studies on preoperative symptoms of depression, anxiety, and CI in patients with glioma, and the pathogenesis is still unclear (Acquaye et al., 2013).

Previous studies shown that the posterior cingulate and the inferolateral parietal lobes in the posterior regions of the default mode network dysfunction, one of the pathophysiological mechanisms of depression, were associated with episodic memory retrieval (Li et al., 2018). In addition, the parietal cortex was one of the commonly identified brain regions of emotional regulation in anxiety disorders using functional MRI, associated with sensory information, with top‐down response‐related information to facilitate flexible, goal‐directed behavior. Meanwhile, prefrontoparietal neural circuits were important in the pathogenesis of anxiety disorders (Wang et al., 2018). Karim et al. (2017) found that severe worry was significantly associated with increased CBF in several neocortical regulatory regions. In our study, the ROIs with significantly different blood flow perfusion changes mainly involved the brain regions, which related to the emotional regulation and cognitive function of the default mode network, affective network, and visual cortical areas (Zeng et al., 2012). The results of our study showed that MMT values in the anterior limb of the internal capsule were significantly increased between the CI group and the CN group, which was consistent with dementia patients (Amen et al., 2017). Previous studies have almost unanimously concluded that the CBF decreases in patients with dementia. Streitparth et al. (2008) found a significant decrease in occipital and temporal CBV values and a significant decrease in CBF values with increasing degree of dementia in the frontal lobe, basal ganglia, and the occipital region, which contradicted to our study in which we found increased values of CBV in the temporal GM. Thus, we suggested that the preoperative psychiatric symptoms in patients with glioma may be related to changes in the perfusion of related brain regions, which may lead to impaired function of fibrous bundles.

There were some differences in the ROIs of depression group, anxiety group, and CI group. Depression group and CI group mainly involved LS, whereas anxiety group involved bilateral ROIs. A previous study showed that increased anxiety was clearly associated with increased CBF, because anxiety increases the sensitivity of CBF to changes in pco_2_ (Van den Bergh et al., 2013). Therefore, we hypothesized that anxiety may be caused by diffuse changes in cerebral perfusion, which could be caused by increased neuronal activity. In addition, the perfusion parameters, including CBV, CBF, MTT, and TTP, represented the cerebral hemodynamics, whereas PS represented the integrity of the blood–brain barrier (BBB). Both abnormalities could cause abnormal WM function, which caused the psycho‐cognitive symptoms (Onishi et al., 2018). Our results showed that the possible mechanism of the depression group and the CI group could be both the changes in cerebral perfusion and BBB, whereas the anxiety group could be only the changes in cerebral perfusion.

With the development of noninvasive cerebral perfusion imaging technology, the different characteristics of tumor perfusion have been used in diagnosis, differential diagnosis, classification, prediction of molecular pathological mutations, monitoring of tumor recurrence, and prediction of generation time in glioma (Karegowda et al., 2017; Onishi et al., 2018; Wang et al., 2021; Zhang et al., 2017). Compared to MRI perfusion imaging, PCT provided absolute quantitative values of the perfusion parameters and the contrast agent concentration with a more linear relationship. However, MRI perfusion could only provide semiquantitative perfusion parameters by comparing an abnormal area with a normal area that may be affected by the tumor (Leiva‐Salinas et al., 2011). Therefore, in our study, PCT was chosen to evaluate the parameters of the tumor and ROIs.

### 4.1 Clinical implications

To our knowledge, this study was the first to analyze the perfusion parameters and preoperative symptoms of depression, anxiety, and CI in patients with glioma. The results of our study were as follows: (1) patients with glioma could have preoperative symptoms of depression, anxiety, and CI; (2) preoperative symptoms of depression, anxiety, and CI in patients with glioma could be closely associated with changes in cerebral perfusion, either on the LS or on the NLS; (3) the presence of tumor could affect cerebral blood perfusion, which could be one of the pathophysiological factors of preoperative psychiatric symptoms in patients with glioma.

### 4.2 Study limitations

Our study had several limitations. First, the samples of this study were relatively small because relatively few patients had PCT scan, which may lead to a possible bias. Second, the hemodynamic parameters were obtained by drawing different ROIs of the brain, so the local occupying effect of the tumor cannot be completely excluded. Third, the subregions in different brain regions could not be analyzed due to the low resolution of PCT. Future studies should replicate this study in larger samples using other advanced neuroimaging techniques to provide additional data on the factors involved in the pathogenesis of psycho‐cognitive symptoms in the patients with glioma.

## 5 CONCLUSION

In conclusion, preoperative symptoms of depression, anxiety, and CI may be present in some glioma patients and may be closely related to the brain perfusion parameters.

## AUTHOR CONTRIBUTIONS

Ke Wang and Wanrui Fu designed the study and wrote the article, Shenjie Li analyzed the image data, Lizhen Chen and Wei Xiang collected the materials, Yajie Gan conducted the psychiatric evaluation, and Ligang Chen and Jie Zhou cared for the patients and revised the report.

## CONFLICT OF INTEREST STATEMENT

The authors declare that they have no conflict of interests related to this work.

### PEER REVIEW

The peer review history for this article is available at https://publons.com/publon/10.1002/brb3.3020.

## Supporting information

Figure S1 The ROIs schematic.

Table S1 The difference in clinical characteristics between the depression group and the non‐depression group.
Table S2 The difference in clinical characteristics between the anxiety group and the non‐anxiety group.
Table S3 The difference in clinical characteristics between CI group and NC group.
Table S4 Correlation between tumor CBV and ROIs perfusion parameters of bilateral brain regions.
Table S5 Correlation between tumor CBF and ROIs perfusion parameters of bilateral brain regions.

## Data Availability

The data supporting the results of this study are available upon request from the corresponding author. The data are not publicly available due to privacy and ethical restrictions.
