# Supplementary figures and images for: Preoperative symptoms of depression, anxiety, and cognitive impairment in glioma patients: A cerebral perfusion CT study
Source: Brain Behav. 2023 May 1;13(6):e3020. doi: 10.1002/brb3.3020 (PMC10275540; doi:10.1002/brb3.3020)

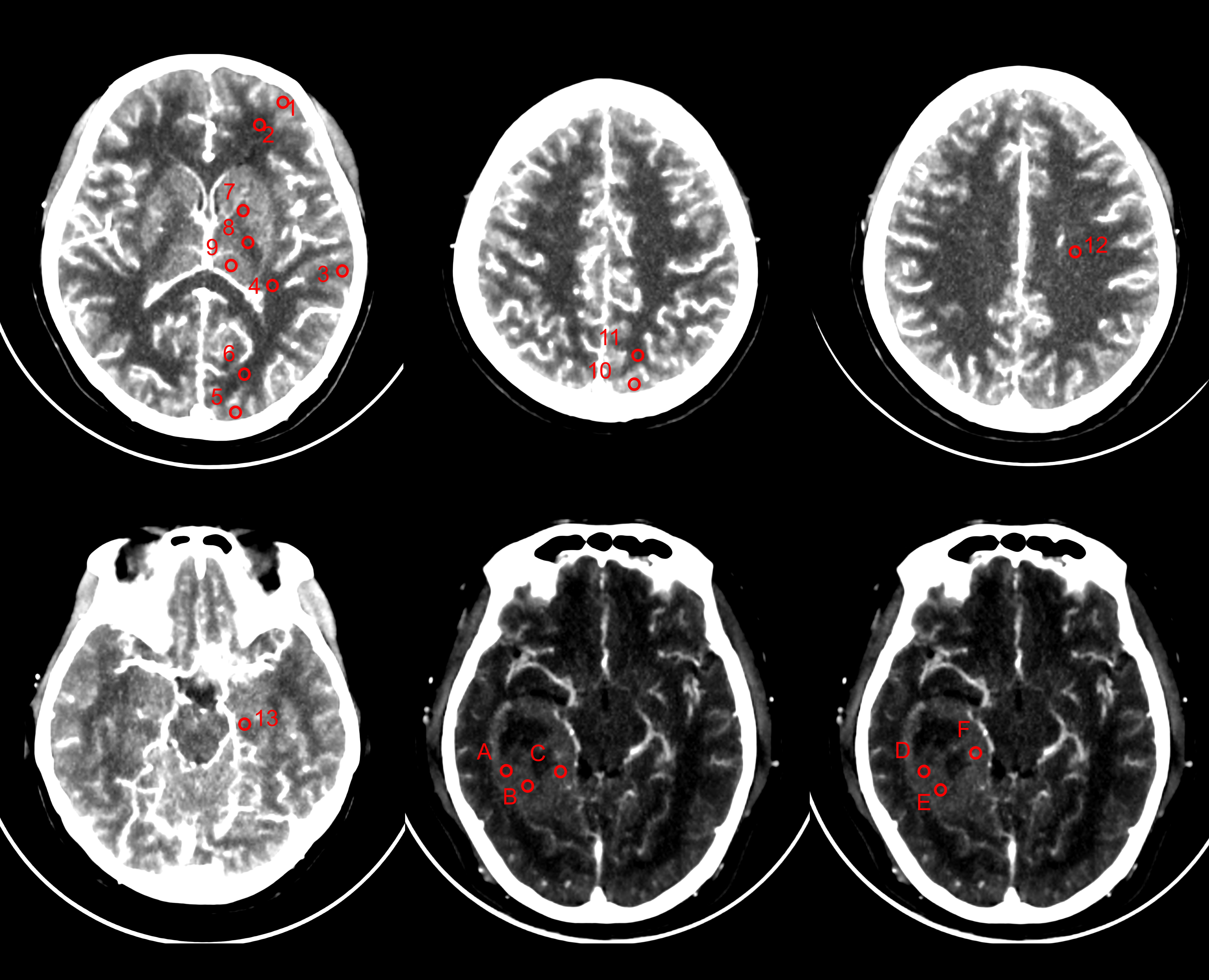

Supplement: Supplementary file 1 — Figure S1 The ROIs schematic. [file BRB3-13-e3020-s002.tif]
