# Supplementary material for: Preoperative symptoms of depression, anxiety, and cognitive impairment in glioma patients: A cerebral perfusion CT study
Source: Brain Behav. 2023 May 1;13(6):e3020. doi: 10.1002/brb3.3020 (PMC10275540; doi:10.1002/brb3.3020)
Supplement: Supplementary file 2 — Table S1 The difference in clinical characteristics between the depression group and the non‐depression group. Table S2 The difference in clinical characteristics between the anxiety group and the non‐anxiety group. Table S3 The difference in clinical characteristics between CI group and NC group. Table S4 Correlation between tumor CBV and ROIs perfusion parameters of bilateral brain regions. Table S5 Correlation between tumor CBF and ROIs perfusion parameters of bilateral brain regions. [file BRB3-13-e3020-s001.docx]

**Supplementary Table 1.** The difference of clinical characteristics between depression group and non-depression group

| Characteristics | Non-depression  (n=21) | Depression  (n=18) | *t* /*χ^2^*/*Z* | *p* |
| --- | --- | --- | --- | --- |
| Age (ys), (Mean ± SD) | 50.19±15.04 | 47.78±14.70 | 0.505^a^ | 0.617 |
| Gender |  |  | 1.293^b^ | 0.256 |
| Male | 12(57.1%) | 7(38.9%) |  |  |
| Female | 9(42.9%) | 11(61.1%) |  |  |
| Tumor grade |  |  | -1.257^c^ | 0.234 |
| WHO I | 2(9.5%) | 1(5.6%) |  |  |
| WHO II | 8(38.1%) | 4(22.2%) |  |  |
| WHO III | 5(23.8%) | 5(27.8%) |  |  |
| WHO IV | 6(28.6%) | 8(44.4%) |  |  |
| Location |  |  | -1.093^c^ | 0.294 |
| Frontal lobe | 10(47.6%) | 5(27.8%) |  |  |
| Temporal lobe | 3(14.3%) | 4(22.2%) |  |  |
| Frontotemporal lobe  (Including insula) | 2(9.5%) | 2(11.1%) |  |  |
| Parietal lobe | 3(14.3%) | 0(0%) |  |  |
| Occipital lobe | 0(0%) | 4(22.2%) |  |  |
| Thalamus and  basic ganglion | 0(0%) | 2(11.1%) |  |  |
| Cerebellum | 3（14.3%） | 1（5.6%） |  |  |
| Side |  |  | -0.624^c^ | 0.587 |
| Right | 9(42.9%) | 7(38.9%) |  |  |
| Left | 11(52.4%) | 8(44.4%) |  |  |
| Bilateral | 1(4.7%) | 3(16.7%) |  |  |
| Education |  |  | -1.002^c^ | 0.379 |
| Primary school | 114(66.7%) | 8(44.4%) |  |  |
| Junior middle school | 2(9.5%) | 6(33.3%) |  |  |
| High school | 3(14.3%) | 2(11.1%) |  |  |
| University and above | 2(9.5%) | 2(11.1%) |  |  |
| Marital status |  |  | 0.215^b^ | 0.643 |
| Married | 2(9.5%) | 1(5.6%) |  |  |
| Unmarried | 19(90.5%) | 17(94.4%) |  |  |
| KPS score |  |  | 0.464^b^ | 0.496 |
| ≤80 | 6(28.6%) | 7(38.6%) |  |  |
| ﹥80 | 15(61.1%) | 11(71.4%) |  |  |
| Note: ^a^ data was analyzed by t-test, ^b^ data was analyzed by Chi-square tests, ^c^ data was analyzed by rank sum test, KPS karnofsky performance status, * *P*＜0.05 | | | | |

**Supplementary Table 2.** The difference of clinical characteristics between anxiety group and non-anxiety group

| Characteristics | Non-anxiety  (n=20) | Anxiety  (n=19) | *t* /*χ^2^*/*Z* | *p* |
| --- | --- | --- | --- | --- |
| Age (ys), (Mean ± SD) | 53.15±12.33 | 44.79±16.12 | 1.825^a^ | 0.076 |
| Gender |  |  | 0.648^b^ | 0.421 |
| Male | 11(55.0%) | 8(42.1%) |  |  |
| Female | 9(45.0%) | 11(57.9%) |  |  |
| Tumor grade |  |  | -0.634^c^ | 0.550 |
| WHO I | 1(5.0%) | 2(10.5%) |  |  |
| WHO II | 6(30.0%) | 6(31.6%) |  |  |
| WHO III | 5(25.0%) | 5(26.3%) |  |  |
| WHO IV | 8(40.0%) | 6(31.6%) |  |  |
| Location |  |  | -1.308^c^ | 0.214 |
| Frontal lobe | 10(50.0%) | 5(26.3%) |  |  |
| Temporal lobe | 3(15.0%) | 4(21.1%) |  |  |
| Frontotemporal lobe  (Including insula) | 1(5.0%) | 3(15.8%) |  |  |
| Parietal lobe | 3(15.0%) | 0(0.0%) |  |  |
| Occipital lobe | 0(2.5%) | 4(21.1%) |  |  |
| Thalamus and  basic ganglion | 1(5.0%) | 1(5.3%) |  |  |
| Cerebellum | 2(10.0%) | 2(10.5%) |  |  |
| Side |  |  | -0.809^c^ | 0.478 |
| Right | 9(45.0%) | 7(36.8%) |  |  |
| Left | 10(50.0%) | 9(47.4%) |  |  |
| Bilateral | 1(5.0%) | 3(15.8%) |  |  |
| Education |  |  | -1.546^c^ | 0.166 |
| Primary school | 14(70.0%) | 8(42.1%) |  |  |
| Junior middle school | 2(10.0%) | 6(31.6%) |  |  |
| High school | 3(15.0%) | 2(10.5%) |  |  |
| University and above | 1(5.0%) | 3(15.8%) |  |  |
| Marital status |  |  | 3.421^b^ | 0.064 |
| Married | 20(100.0%) | 16(84.2%) |  |  |
| Unmarried | 0(0.0%) | 3(15.8%) |  |  |
| KPS score |  |  | 0.205^b^ | 0.651 |
| ≤80 | 6(30.0%) | 7(36.8%) |  |  |
| ﹥80 | 14(70.0%) | 12(63.2%) |  |  |
| Note: ^a^ data was analyzed by t-test, ^b^ data was analyzed by Chi-square tests, ^c^ data was analyzed by rank sum test, KPS karnofsky performance status, * *P*＜0.05 | | | | |

**Supplementary Table 3.** The difference of clinical characteristics between CI group and CN group

| Characteristics | NC  (n=29) | CN  (n=10) | *t* /*χ^2^*/*Z* | *p* |
| --- | --- | --- | --- | --- |
| Age (ys), (Mean ± SD) | 48.17±16.12 | 51.70±9.89 | -0.648^a^ | 0.521 |
| Gender |  |  | 0.009^b^ | 0.925 |
| Male | 14(48.3%) | 5(50%) |  |  |
| Female | 16(51.7%) | 5(50%) |  |  |
| Tumor grade |  |  | -1.603^c^ | 0.128 |
| WHO I | 2(6.9%) | 0(10.0%) |  |  |
| WHO II | 11(37.9%) | 1(10%) |  |  |
| WHO III | 8(27.6%) | 2(20%) |  |  |
| WHO IV | 8(27.6%) | 6(60.0%) |  |  |
| Location |  |  | -0.316^c^ | 0.764 |
| Frontal lobe | 10(34.5%) | 5(50.0%) |  |  |
| Temporal lobe | 6(20.7%) | 1(10.0%) |  |  |
| Frontotemporal lobe  (Including insula) | 4(13.8%) | 0(0.0%) |  |  |
| Parietal lobe | 2(6.9%) | 1(10.0%) |  |  |
| Occipital lobe | 3(10.3%) | 1(10.0%) |  |  |
| Thalamus and  basic ganglion | 1(3.5%) | 1(10.0%) |  |  |
| Cerebellum | 3(10.3%)) | 1(10.0%) |  |  |
| Side |  |  | -1.710 ^c^ | 0.128 |
| Right | 14(48.3%) | 2(20.0%) |  |  |
| Left | 13(44.8%) | 6(60.0%) |  |  |
| Bilateral | 2(6.9%) | 2(20.0%) |  |  |
| Education |  |  | -0.661^c^ | 0.558 |
| Primary school | 17(58.6%) | 5(50.0%) |  |  |
| Junior middle school | 6(20.7%) | 2(20.0%) |  |  |
| High school | 4(13.8%) | 1(10.0%) |  |  |
| University and above | 2(6.9%) | 2(20.0%) |  |  |
| Marital status |  |  | 1.121^b^ | 0.290 |
| Married | 26(89.7%) | 10(100.0%) |  |  |
| Unmarried | 3(10.3%) | 0(0.0%) |  |  |
| KPS score |  |  | 1.681^b^ | 0.195 |
| ≤80 | 8(27.6%) | 5(50.0%) |  |  |
| ﹥80 | 21(72.4%) | 5(50.0%) |  |  |

Note: CI cognitive impairment, CN cognitive normality, ^a^ data was analyzed by t-test, ^b^ data was analyzed by Chi-square tests, ^c^ data was analyzed by rank sum test, KPS karnofsky performance status, * *P*＜0.05

**Supplementary Table 4.** Correlation between tumor CBV and ROIs perfusion parameters of bilateral brain regions

| ROIs | Parameter | *rs* | *P* |
| --- | --- | --- | --- |
| LS occipital GM | CBV | 0.425 | 0.017* |
|  | MTT | 0.358 | 0.048* |
|  | TTP | 0.428 | 0.016* |
| LS anterior limb of internal capsule | TTP | 0.364 | 0.044* |
| LS hippocampus | CBV | 0.435 | 0.013* |
|  | MTT | 0.424 | 0.016* |
|  | TTP | 0.352 | 0.048* |
| LS occipital WM | CBV | 0.487 | 0.007* |
|  | TTP | 0.417 | 0.024* |
| NLS temporal GM | CBV | 0.340 | 0.043* |
| NLS anterior limb of internal capsule | CBV | 0.384 | 0.025* |
|  | MTT | 0.370 | 0.031* |
|  | TTP | 0.425 | 0.012* |
| NLS postterior limb of internal capsule | CBV | 0.397 | 0.016* |
|  | MTT | 0.337 | 0.045* |
|  | TTP | 0.343 | 0.041* |
| NLS hippocampus | CBV | 0.342 | 0.041* |
|  | MTT | 0.375 | 0.024* |
| NLS parietal GM | TTP | 0.365 | 0.029* |
| NLS occipital WM | MTT | 0.341 | 0.045* |
|  | TTP | 0.416 | 0.013* |
| NLS parietal WM | MTT | 0.388 | 0.021* |

Note: ROIs regions of interest, LS lesion side, NLS non-lesion side, GM gray matter, WM white matter, CBV cerebral blood volume, TTP time to peak, MTT mean transition time, * *P*＜0.05,

**Supplementary Table 5.** Correlation between tumor CBF and ROIs perfusion parameters of bilateral brain regions

| ROIs | Parameter | *rs* | *P* |
| --- | --- | --- | --- |
| LS temporal GM | MTT | -0.405 | 0.024* |
| LS anterior limb of internal capsule | MTT | -0.409 | 0.022* |
| LS parietal GM | CBF | 0.356 | 0.046* |
| LS occipital WM | CBV | -0.419 | 0.017* |
| NLS parietal WM | MTT | -0.332 | 0.048* |

Note: ROIs regions of interest, LS lesion side, NLS non-lesion side, GM gray matter, WM white matter, CBV cerebral blood volume, CBF cerebral blood flow, MTT mean transition time, * *P*＜0.05,
